# Editorialets

**Published:** 1909-03

**Authors:** 


### Editorialets.

Dr. T. B. Bass, late first assistant physician to the Epileptic Colony at Abilene, having succeeded Dr. John Preston (transferred to [Austin] State Insane Asylum, vice Worsham, resigned) as Superintendent. Dr. M. M. Carrick of Dallas has been appointed to succeed Dr. Bass as first assistant physician. Good appointment.

Wanted—Slightly used instruments and all kinds of office equipment, in good condition. Fair prices for reliable goods. Distance no object. Write Henderson, 127 East 23d St., New York.

Free Sample of a new patent obstetrical examination cot will be sent to physicians sending card or prescription blank. Other novelties. Address Medical Equipment Company, 127 East 23d St., New York.

A Remarkable Case of Amnesia.—Dr. Simmons does not remember the time and circumstances when he was conducting a medical institute and water cure with homeopathy and liquid oxygen on the side, at Lincoln, Nebraska, twenty years ago. See his letter to Lydston in this issue. The cut accompanying Lydston's article, it is hoped, will refresh his memory.

Clean at Last.—The *Texas State Journal of Medicine* (January number, p. 216) gleefully announces that it has dropped the l—a—s—t one of the "unclean and infectious" ads. it admits that it has been carrying, and that hereafter it will be clean. Congrats. This seems to have been a case of kettle and pot, or mote and beam, all along.

It is hardly to be expected that a monthly can give the latest news, but the "Red Back" is usually the first to give happenings of importance to the doctors of Texas. The following will be news to most of them.

City Health Officer Cox of Galveston has caused to be introduced in the Legislature a bill creating a colony for lepers, a number of cases having been found in Galveston.

The Legislature will adjourn soon. The Governor will immediately call an extra session. But the extra session can consider only such matters as are laid before it by the Governor. This will be, first, the great appropriation bill, which will not be reached this session. Second, certain "platform demands" as yet untouched. It is feared that many reform measures, badly needed, will have to go over, two years, such, at least, as are not disposed of in the next few days, this being March 1st.

---

THE TUBERCULOSIS SANITARIUM BILL has passed the Senate with good prospects of passing the House this week. The State Board of Health bill is still slumbering.

---

THE CASTRATION BILL (Walter, of Gonzales—House Bill) went through the House with a rush—78 to 23. It was put on final passage under suspension of rules and passed. If reached, it is thought it will meet little or no opposition in the Senate. The bill provides for penitentiary sentence for assault to rape by violence, two to fifteen years, and castration by the prison physician. Texas will be the first State to pass such a law, if it is passed.

---

THE BILL TO CREATE A CHAIR OF HOMEOPATHY in the Medical Department of the State University passed the Senate so amended (by Masterson) as to defeat it. That is, the creation of such chair is to be left to the discretion of the Board of Regents. Well, with two representative orthodox physicians on that Board—well, hardly!

---

THE BILL TO EXEMPT GRADUATES of the State Medical College from examination by the piebald, Joseph's coat, crazy-quilt board was really killed by rival (private) medical schools—all of which wanted the same kind of exemption.

---

THE ANTI FIRE CRACKER AND TOY PISTOL BILL is still hanging fire. I'm afraid it will be a flash in the pan (no pun intended).

---

BRUMBY SAYS there is smallpox in over one hundred counties in Texas. And yet the cranks at Fort Worth tried—unsuccessfuly—to get an injunction to prevent vaccination of school children.

---

DR. R. W. SKIPPER, of Lovelady, and his son, Dr. C. W. Skipper, of Abilene, have removed to Port Arthur, Texas, to engage in general practice. Success to them.

---

DR. AIME PAUL HEINECK'S valuable paper on "Acute Traumatic Tetanus, Treated by Magnetism Sulphate," will appear in April number of the "Red Back."

DR. J. H. MCCALEB, of Gonzales, Texas, died recently at San Antonio, Texas, after an operation for appendicitis. He was a prominent, well known and popular physician, active in Association work. It is sad to recall that his brother, Dr. G. W. McCaleb, who went with him to Gonzales, died about a year ago. Another brother, Dr. W. E. McCaleb, survives them, and is practicing at Webberville, Texas. Dr. J. H. McCaleb was a prominent Mason, and was recently elected Generalissimo of Gonzales Commandery, K. T.

---

"VACCINATION" AGAINST TYPHOID FEVER.—A board of medical experts met at the War Department, December 7th, to consider the plan or method originated by Sir Almott Wright of inoculating against typhoid. They decided that it is useful and harmless, and recommended that the practice of voluntary "vaccination" [inoculation?] be introduced in the U. S. army. The board consisted of Surgeon General U. S. A. O'Reilly, Dr. V. C. Vaughan (Ann Arbor), Dr. W. T. Councilman (Harvard), Dr. Jno. H. Musser, U. of P., Dr. Alexander Lambert (N. Y.), Dr. Simon Flexner (Rockefeller Institute), and Dr. W. S. Thayer (Johns Hopkins).

---

FLEXNER'S SERUM, which has been so efficacious in the cure of epidemic cerebro-spinal meningitis, is now being manufactured in the laboratory of Dr. George Dock at the Medical Department of Tulane University. It will be supplied to the profession of Louisiana and no doubt of all neighboring States.

---

I DESIRE to say that if any of my delinquent subscribers wish to pay, and not be dropped, and are unable to do so, I will gladly indulge them if they will only *say so*. A p. c. will be sufficient.

---

DR. L. B. ROEBUCK has removed from Anson, Texas, to Hawley, Texas.

---

DR. W. W. LUNN, of Houston, died February 23 (ult.), from the effects of a septic wound of the finger, inflicted while he was operating, some two years ago. We greatly regret his death. Dr. Lunn was one of the foremost and popular surgeons of the Southwest. He was a graduate of Tulane, class of 1884. His son, Dr. E. R. Lunn, who was associated with him, will still conduct the Lunn Sanitarium, founded by his father many years ago.

---

DR. GEORGE MACDUFF ANDERSON, of Tanglewood, says: "I see the Osteos and Eclectics and Physio fellows want a chair. How would you look leading your 'Tame tubercle' by a string with one hand and one of these fellows with the other? Go on with the

'Chronicles'; cut off his 'tentacles.' What are we coming to, anyway?"

---

"I HOLD in high appreciation your most valuable and exhaustive write-up of the 'Octopus.' The 'Red Back' grows in value and importance as it advances in years."--SAM R. BURROUGHS.

---

"COMPLIMENT YOU on your 'Chronicles of the Octopus.' It is most excellent; I may say a masterstroke of a masterpen."—DR. C. S. VENABLES, San Antonio.

---

TIME-TRIED and fire-tested old "Red Back."—W. D. COLLINS.

---

DR. T. H. HARRELL has removed from Willis to Smiley, Texas

---

DR. W. B. COLLINS says "Long live the 'Red Back' and its worthy editor,—time-tried and fire-tested."

---

DR. J. M. TOWNES renews for the twenty-fifth and twenty-sixth year, and says "Can't do without it."

---

DR. G. B. FOSCUE, to whom more than to any one else, perhaps, we are indebted for the patch work crazy-quilt Mixed Board, has resigned in disgust from that board. Dr. James D. Osborn, ex-President Texas State Medical Association, is now President of the Board. Dr. Foscue, also ex-President Texas State Medical Association, was its President one term and its first Secretary. Dr. R. H. McLeod, of Palestine, has been appointed to fill the vacancy thus created.

---

"I AM GLAD to have found one editor with the courage to speak his convictions and *tell the truth.* Your 'Chronicles of the Octopus' was a solar-plexus blow straight from the shoulder."—DR. J. C. DENSTEN, Scranton, Penn.

